# Epigenetic Modulation of Microglia Function and Phenotypes in Neurodegenerative Diseases

**DOI:** 10.1155/2021/9912686

**Published:** 2021-05-29

**Authors:** Li Wang, Chao-Chao Yu, Xin-Yuan Liu, Xiao-Ni Deng, Qing Tian, Yan-Jun Du

**Affiliations:** ^1^College of Acupuncture and Orthopedics, Hubei University of Chinese Medicine, Wuhan, Hubei, China; ^2^Department of Tuina, Shenzhen Traditional Chinese Medicine Hospital, Shenzhen, China; ^3^The Fourth Clinical College, Guangzhou University of Chinese Medicine, Shenzhen, China; ^4^Department of Pathology and Pathophysiology, School of Basic Medicine, Tongji Medical College, Huazhong University of Science and Technology, Wuhan, China

## Abstract

Microglia-mediated neuroinflammation is one of the most remarkable hallmarks of neurodegenerative diseases (NDDs), including AD, PD, and ALS. Accumulating evidence indicates that microglia play both neuroprotective and detrimental roles in the onset and progression of NDDs. Yet, the specific mechanisms of action surrounding microglia are not clear. Modulation of microglia function and phenotypes appears to be a potential strategy to reverse NDDs. Until recently, research into the epigenetic mechanisms of diseases has been gradually developed, making it possible to elucidate the molecular mechanisms underlying the epigenetic regulation of microglia in NDDs. This review highlights the function and phenotypes of microglia, elucidates the relationship between microglia, epigenetic modifications, and NDDs, as well as the possible mechanisms underlying the epigenetic modulation of microglia in NDDs with a focus on potential intervention strategies.

## 1. Introduction

Neurodegenerative diseases (NDDs) are common chronic neurological disorders with features of progressive neuronal loss within the central nervous system (CNS) resulting in gradual deterioration of motor symptoms and/or cognitive function. Of these, Parkinson's disease (PD), Alzheimer's disease (AD), and Amyotrophic lateral sclerosis (ALS) are the main typical diseases [1–3]. AD is one of the most common NDDs, characterized by amyloid-*β* (A*β*) plaques and neurofibrillary tangles (NFTs), leading to cognitive deficits [4]. PD is distinguished by progressive and selective loss of dopaminergic (DA) neurons in the substantia nigra pars compacta (SNpc) [5], while ALS manifests as degeneration and loss of motor neurons (MNs) [6]. These pathologies are mostly correlated with protein misfolding and aggregation, such as A*β* and tau in AD, *α*-synuclein (SNCA) in PD, and TAR DNA-binding protein 43 (TDP-43) in ALS [7]. NDDs are affecting a massive and ever-increasing population worldwide, resulting in severe inconvenience to patients in their daily lifespan and imposing a heavy burden on public health and society [8]. Unfortunately, the mechanisms of NDDs are still poorly understood, and no ideal drugs are available to slow down the onset and progression of NDDs. It is thus essential to gain insight into the pathogenesis of NDDs and to seek effective treatments.

In recent studies, accumulating evidence has underlined the core role of the immune system in the onset and progression of NDDs [9, 10]. Existing and emerging data highlight the importance of microglia in health and diseases [11]. Microglia are the predominant type of glial cells, constituting approximately 5-12% of the total population of CNS cells, which are mostly expressed in brain areas such as the cortex and hippocampus [12, 13]. As the crucial immune cells of the brain, microglia originate from myeloid precursors and migrate to the brain in the early state of embryonic development, with an essential role in maintaining normal development of the brain and environmental homeostasis of the CNS [14]. Conversely, uncontrolled or aberrantly activated microglia potentially trigger brain diseases. Accumulating evidence has suggested that microglia are implicated in the onset and exacerbation of NDDs [15, 16]. Moreover, the role of microglia in NDDs depends on its phenotypes. Consistent with this, microglia play a “double-edged sword” role in NDDs [17]. In the pathological process of NDDs, there are both activated microglia and increased release of inflammatory mediators [18, 19]. Neuroinflammation has become a common pathological hallmark of NDDs, and microglia are the prime effectors in the regulation of inflammatory response [20]. Yet, the specific mechanisms of action surrounding microglia are far from clarified. Therefore, modulating the activation or phenotypes of microglia to attenuate inflammatory response is recognized as an attractive target in the therapeutic of NDDs.

Epigenetic modifications are closely associated with various diseases, including NDDs, tumors, and autoimmune diseases, which mainly affect complex processes such as memory, motor, and cognitive function in the brain [21–24]. Recent efforts have focused on elucidating the effects of epigenetic modifications in regulating microglia under physiological and pathological states, which have been proven to be feasible [25, 26]. A study has revealed the possible molecular pathways for regulating the function and phenotypes of microglia from the perspective of transcriptome and epigenetic, which provides basis for the treatment of NDDs [26]. The investigation of epigenetic modifications regulating the function and phenotypes of microglia still needs to be further validated.

Given the reversibility of epigenetic and the role of microglia in NDDs, this review mainly focuses on the function and phenotypes of microglia, elucidates the relationship between microglia, epigenetic modifications, and NDDs, as well as the possible mechanisms underlying the epigenetic modulation of microglia in NDDs.

## 2. Microglia: Function and Phenotypes

### 2.1. Microglia and Its Function

Microglia, the antigen-presenting phagocytes of CNS, with their continuous surveillance of the intracerebral microenvironment in highly motile processes and their plasticity and transcriptional potency enable them to exert their major roles as sentinels and early responders when the brain is invaded by pathogens [27]. Under normal physiological circumstances, microglia respond to noxious stimuli and are involved in tissue repair, injury healing, clearance of necrotic neurons, and engulfment of cellular debris, as well as mediating synaptic pruning, emerging as key players in the formation of mature neuronal circuits and maintenance of homeostasis in the cerebrum [20, 28–30], while injury, infection, NDDs, or other situations linked to imbalances in the homeostatic state of the brain trigger alterations in microglia morphology, function, and gene expression ocurred, which is generally termed as “activation” [31–33]. In resting conditions, microglia are seemingly ramified in morphology, characterized by active protrusions and dynamic exploration of the circumambient microenvironment. Once activated, aberrant microglia gradually transform into a motile amoeboid form accompanied by the release of variable cellular products, including chemokines, proinflammatory cytokines, and lipid mediators [34, 35]. Microglia can promote the expression of anti-inflammatory and neurotrophic factors, scavenging cellular debris and facilitating nerve repair. However, microglia also secrete proinflammatory cytokines and cytotoxic mediators, triggering a cascade of inflammatory responses [20]. Due to their heterogeneity and perception of changes in the intracerebral microenvironment, microglia are activated and polarized into two distinct phenotypes, namely, M1 and M2 phenotypes.

### 2.2. Different Polarization Phenotypes of Microglia

The “classically activated” M1 phenotype, also known as the proinflammatory M1 phenotype, is activated primarily by lipopolysaccharides (LPS) or interferon-*γ* (INF-*γ*), releasing a series of proinflammatory cytokines or mediators, such as TNF-*α*, IL-1*β*, IL-6, and iNOS, and secreting large amounts of neurotoxic factors, resulting in synaptic loss and neurotoxic injury, together with the induction of inflammatory response [36]. Of these, CD86 and iNOS are the main surface markers of this phenotype [37, 38]. Meanwhile, the “alternatively activated” M2 phenotype, also termed as the anti-inflammatory M2 phenotype, is activated under the stimulation of IL-4 or IL-13 and secretes neurotrophic factors such as BDNF, VEGF, and IGF-1, as well as anti-inflammatory cytokines including TGF-*β* and IL-10, which exerts neuroprotective effect via facilitating tissue repair and resolving inflammation [36, 39]. Meanwhile, the M2 microglia phenotype induces the expression of key specific markers such as CD206 and arginase-1 (Arg1) [40].

Activated microglia exerting both detrimental and beneficial effects on neurons which may be attributed to the function and polarization status of microglia after neuronal injury [41]. It has been shown that there is a potential interconversion between distinct phenotypes of microglia in the complex intracerebral microenvironment, which indicates that M1/M2 phenotypes are in a dynamic state [42, 43]. Microglia are extremely plastic and capable of switching their phenotypes in accordance with their role. Further research is being carried out on the regulation mechanisms of microglia phenotypes and function in diseases.

## 3. Microglia-Mediated Neuroinflammation in the Neurodegenerative Diseases

The wide-ranging pathogenic mechanisms of NDDs are poorly understood, which pose tremendous challenges for drug development. Neuroinflammation, a fundamental immune response within the CNS caused by a variety of pathological injuries, is regarded as the most prominent hallmark of NDDs, including AD, PD, and ALS [44, 45]. With progressive studies in recent years, there is a revolutionary understanding of microglia, including their role in the physiology and pathology of NDDs. Mounting evidence suggests that microglia-mediated neuroinflammation contributes to the onset and progression of NDDs [20, 46].

As the major source of proinflammatory factors, microglia represent pivotal mediators of neuroinflammation, are involved in various aspects of neuroinflammation, and trigger or mediate multiple cellular responses [47–49]. It has been well established that microglia are activated and trigger persistently inflammatory response, leading to progressive neuronal loss or damage in NDDs [20, 50]. For instance, in the early pathology of AD, microglia activation is implicated in the clearance of A*β*. With the progression of AD, the aberrantly activated microglia enhance the expression of proinflammatory cytokines such as IL-1*β*, IL-6, and TNF-*α*, triggering increased A*β* accumulation and neuroinflammation [51]. Studies have confirmed that curcumin can suppress the TLR4/NF-*κ*B pathway and downregulate the expression of TREM2 in BV-2 cells, thereby significantly regulating microglia M1/M2 polarization and reducing inflammatory response [48]. Meanwhile, aberrantly activated microglia and prolonged neuroinflammation contribute to the progressive death of DA neurons in PD [52, 53]. Yao et al. [54] have reported that FTY720 reduces the expression of ROS by inhibiting the PI3K/AKT/GSK-3*β* signaling pathway and decreasing phosphorylation of p65, effectively inhibiting the activation of NLRP3, ultimately reducing neuronal injuries induced by microglia activation, thus ameliorating progression of PD. NLRP3 inflammasome serves as a key mediator of the deleterious action of microglia, while its deletion blocks alterations of microglia morphology and protects the brain from toxic substances [55]. In addition, studies have shown that there exist activated microglia and inflammation in the ALS [56, 57]. Collectively, microglia-mediated neuroinflammation is emerging as a key player in the pathological progression of NDDs. Certainly, identifying the pathways or molecular mechanisms of microglia-mediated neuroinflammation is of utmost importance, and future exploratory studies in this area warrant validation.

Taken together, microglia may exert dual actions in NDDs, depending largely on its function and phenotypes. Indeed, inappropriate or aberrant activation of microglia induces a series of deleterious effects, exacerbating the progression of NDDs pathologies. Microglia-mediated neuroinflammation constitutes the possible mechanisms for the onset or deterioration of NDDs. Therefore, modulation of microglia function and phenotypes for homeostasis may be an effective therapeutic method to alleviate the deterioration of NDDs. Multiomic technologies, such as proteomics, metabolomics, and epigenetics, have made it possible to identify the signature of microglia and modulate their phenotypes.

## 4. Epigenetic in Neurodegenerative Disorders

Recent years have witnessed a rapid development in epigenetic. In the last decade, epigenetic has embarked on tremendous implications in the fields of cognitive function, stem cell aging, neuroplasticity, and psychopathology [58–61]. Epigenetic is the process of regulating gene expression via altering their transcriptional activity other than the genome, with the main mechanisms including DNA methylation and histone modifications, as well as noncoding RNAs [62]. Epigenetic modifications are of great significance to human health, and its dysfunctions play a causative role in various NDDs, including AD, PD, and ALS [23, 63, 64].

Not surprisingly, alterations in DNA methylation are closely associated with aging, particularly in AD and PD [65–67]. Intriguingly, in the postmortem cortex of AD patients, the remarkable increase in the expression of PSEN1 is linked to reduced methylation both at CpG and non-CpG sites, contributing to the worsening of AD pathological features [68]. DNMT1 and DNMT3A are important DNA methyltransferases that have major roles in brain function during adulthood. In the targeted double knockout mice of DNMT1 and DNMT3A, synapses in the CA1 region of the hippocampus exhibit loss of long-term potentiation (LTP), along with deficits in learning and memory [69]. A subsequent study also revealed that the hippocampal region of postmortem AD samples exhibited global DNA hypomethylation along with a decline in the expression of DNMT1 and DNMT3A [70]. Moreover, studies have confirmed that levels of DNA methylation are inversely correlated with A*β* and NFTs in the hippocampus, implying the pivotal role for DNA methylation in the pathological progression of AD [71, 72]. The aggregation of SNCA is well established as a contribution to the pathogenesis of PD [73]. Current studies have showed marked hypomethylation in postmortem brain tissue and blood samples from PD patients, which is correlated with the risk gene of SNCA variability [74, 75]. Consistent with this, it was observed that reduced levels of DNMT1 were involved in SNCA, resulting in DNA hypomethylation in PD models [76]. Genome-wide methylation analysis of PD patients has revealed that DNA methylation is closely correlated with the progression of PD [77]. Furthermore, aberrant DNA methylation may also be a new direction in the pathogenesis of ALS [78, 79]. Taken in all, it is clear that DNA methylation plays a crucial role in the pathogenesis of NDDs, and studies on pharmacological interventions in NDDs from the perspective of DNA methylation are yet to be validated.

There is an evidence that histone modifications play a critical role in NDDs, and histone acetylation represents one of the most studies [80]. Dysregulated histone acetylation is implicated in multiple pathways of NDDs, including apoptosis [81], inflammatory response [82], neuronal plasticity, and cognition [83, 84]. It is increasingly clear that histone acetylation is involved in the etiology of AD. Subsequent studies have shown that histone acetylation levels are remarkably reduced in both animal models and postmortem brains of AD [85, 86]. These alterations in histone acetylation contribute to cognitive impairment [87], while selective inhibition of histone deacetylase 2 (HDAC2) is potent in maintaining the homeostasis of histone acetylation and reversing cognitive deficits [88]. Furthermore, a genome-wide analysis has identified 4162 differential acetylated variant peaks between AD cases and controls, and these differences are relevant to the pathology of A*β* and tau [89]. In addition, studies have shown that histone acetylation also has a major role in the pathogenesis of PD [90, 91]. Park et al. [91] have shown that histone acetylation levels of midbrain DA neurons are distinctly higher in PD patients than those in the control individuals. Several studies have also shown that HDAC inhibitors can inhibit the neurotoxicity induced by 1-methyl-4-phenylpyridinium (MPP+) and SNCA in PD [90, 92]. Meanwhile, various inhibitors of HDACs have been proven to exert neuroprotective actions in NDDs [93, 94]. Overall, dysregulation of histone acetylation plays a causative role in the progression of PD, and maintaining homeostasis of histone acetylation may have therapeutic potential in NDDs.

Moreover, dysregulation of noncoding RNAs is associated with the pathogenesis of NDDs, of which microRNAs are the most broadly studied. Several lines of evidence link AD to microRNA dysregulation. For instance, BACE1 is known as one of the APP-cleaving enzymes of A*β* production, and its expression is increased in sporadic AD [95]. By exploring changes in microRNA expression profiles, Hébert et al. discovered that the miR-29a/b-1 cluster inhibited the expression of endogenous BACE1 and was markedly reduced in sporadic AD [96]. Meanwhile, a recent study has revealed that addition of microRNA-34a-5p or microRNA-125b-5p attenuates A*β*-induced neurotoxicity via targeting BACE1 [97]. Additionally, Li et al. have unveiled that overexpression of microRNA-219-5p contributes to tau phosphorylation in brain tissue from AD patients [98]. These findings highlight the paramount role of microRNAs in the production of A*β* and the formation of NFTs. Similarly, microRNAs are also implicated in the etiology of PD. Doxakis has shown that miR-7 and miR-153 modulate levels of SNCA and are involved in the pathophysiological process of PD [99]. More recently, miR-155 exerts a major role in inflammation triggered by SNCA, and blockade of miR-155 reduces SNCA-induced neurotoxicity in PD mice [100]. Also, microRNAs appear to have a significant role in the pathophysiology of ALS [101]. These studies support the concept that noncoding RNAs play a vital role in NDDs, and therefore identifying specific types of noncoding RNAs or targeting pathways may be a potential solution.

Overall, epigenetic plays a crucial role in the occurrence and progression of NDDs via multiple signalling pathways or molecular mechanisms. Additional studies should be conducted with the aim of grasping the specific pathological mechanisms of epigenetic in these diseases and providing targeted therapeutic strategies.

## 5. Epigenetic Regulation of Microglia Function and Phenotypes in Neurodegenerative Diseases

Genetic correlation research has unveiled a tight link between microglia genes and NDDs [102]. Indeed, microglia will exhibit different transcriptional signatures in accordance with their phenotypes, confirming the plasticity and complexity of microglia [103]. There is an increasing evidence now linking epigenetic to the regulation of microglia function and phenotypes in NDDs [26, 104]. Meanwhile, RNA sequencing has revealed distinct transcriptome signatures within microglia that facilitate the homeostasis of intracerebral environment and regulation of immune responses [105]. Although studies have confirmed the actions of epigenetic in microglia, the definite molecular mechanisms underlying epigenetic regulation of microglia in NDDs remain to be elucidated [106, 107]. Therefore, it is imperative to delve into the molecular mechanisms underlying the epigenetic regulation of microglia function and phenotypes in NDDs, from DNA methylation to diverse histone modifications, as well as non-coding RNAs, so as to seek potential targets for the treatment of NDDs.

### 5.1. DNA Methylation

DNA methylation is a known mode of epigenetic modifications. The process involves the C5 position of cytosine-guanine dinucleotide (CpG) being covalently linked to a methyl group in the presence of DNA methylation transferases, culminating in the formation of 5-methylcytosine [108]. These methyl groups are added to gene promoters by a family of DNA methyltransferases (DNMTs), including DNMT1, DNMT3A, and DNMT3B [108]. CpG is the dominant site of DNA methylation. And the CpG-rich regions, known as the CpG islands, are mainly situated upstream of the gene promoter region/transcription initiation site and are usually unmethylated, allowing the expression of genes [109]. DNA methylation may block the binding of polymerases or transcription factors (TFs), both of which inhibit gene expression, generally described as silent gene expression [71]. There is a substantial evidence that DNA methylation has a significant role in the aberrant expression of genes linked to NDDs [67, 68, 74, 75, 78]. In addition, aberrant DNA methylation facilitates the activation of microglia and the secretion of proinflammatory cytokines, leading to deterioration in the pathological process of NDDs [110].

The triggering receptor expressed on myeloid cells 2 (TREM2) is predominantly expressed on the membranes of microglia in the CNS, and its variants have been recognized as risk factors for NDDs [111]. Several studies have demonstrated the regulation of gene expression by DNA methylation in microglia, including TREM2 and BACE1 [112, 113]. Indeed, BACE1 inhibitors may reduce the production of A*β* and facilitate the neuroprotective action of microglia in AD [95, 114]. Likewise, it was shown that SAH (DMNT inhibitor) induced hypomethylation of the presenilin 1 (PSEN1) promoter regions, along with an increase in A*β* aggregation in BV-2 microglia [115]. IL-1*β*, one of the vital cytokines secreted by activated microglia, is a crucial mediator of the inflammatory response, and its elevated expression is correlated with aging or tau-induced cognitive decline [116]. Sirtuin 1 (SIRT1), a histone deacetylase, is implicated in senescence and inflammation [117, 118]. Cho et al. [119] have reported that targeted activation of IL-1*β* transcription by microglia SIRT1 deletion is probably modulated by hypomethylation of specific CpG sites within the proximal promoter of IL-1*β*, resulting in cognitive deficits in two separate models of aging, indicating that altered methylation of IL-1*β* may be associated with cognition. Matt et al. [120] have shown that reduced methylation of the IL-1*β* gene promoter in primary microglia is implicated in elevated IL-1*β* mRNA and long-lasting sickness behavior in aged mice, indicating that DNA methylation facilitates activation of microglia. Furthermore, particularly in PD [121] and ALS [122], altered DNA methylation has been shown to have a major impact on the phenotypes and function of microglia.

Taken together, DNA methylation in microglia seems to be altered in various NDDs, which has vast implications for the regulation of microglia function and phenotypes. The available DNA methylation studies provide preliminary insights into microglia, with more specific gene transcription or molecular mechanisms remaining to be refined.

### 5.2. Histone Modifications

Histones and nuclear proteins are the main protein components of chromatin that are wrapped by DNA to form the structure of the nucleosome. They are classified into five main categories, including H1/H5, H2A, H2B, H3, and H4. H2A, H2B, H3, and H4 are the core histones, which exist as dimers and constitute the octamer that the DNA strand enwraps. Histones may exhibit modifications such as acetylation, methylation, ubiquitination, phosphorylation, sumoylation, adenylation, and glycosylation [123]. According to distinct types or sites of modifications, histones can modify the structure of DNA, arranging it into heterochromatin or euchromatin and ultimately inhibiting or activating the expression of that gene. Studies have shown that histone modifications are closely interrelated with the pathology of NDDs [101, 124]. Of these, the action of histone acetylation in NDDs has been extensively studied [67].

#### 5.2.1. Histone Acetylation

Histone acetylation consists of acetylation and deacetylation. Acetylation of histone tails with lysine can neutralize the positive electrical charges on these residues, causing a reduction in the affinity between local DNA and histones, as well as loosening chromatin structure, facilitating the binding of TFs to DNA, thus activating gene transcription. Conversely, histone deacetylation is the removal of acetyl groups from histone tails followed by tight chromatin structure and inhibition of gene transcription [125]. The state of histone acetylation is mainly determined by 2 opposing types of enzymes and their activities, namely, histone acetyltransferases (HATs) and histone deacetylases (HDACs). Both HATs and HDACs are of paramount significance in maintaining homeostasis of histone acetylation.

A handful of recent studies has shown that histone modifications have an essential role in the onset and progression of NDDs. The role of HDACs in the regulation of microglia has come into focus. Datta et al. [126] have shown that deletion of HDAC1and HDAC2 promotes microglia phagocytosis of A*β* and improves cognition in a mouse model of AD, implying the vital role of HDAC1 and HDAC2 in the maintenance of microglia function. A subsequent study has revealed that microglia activation mediates overexpression of HDAC2, reduces levels of histone acetylation, and suppresses transcription and expression of BDNF and c-fos, leading to memory impairment. Then, injection of the adenoassociated virus (ShHDAC2) in the dorsal hippocampus attenuates microglia activation and effectively reversed these pathological processes [127]. Zhu et al. [128] have shown that overexpression of HDAC3 promotes A*β* levels and microglia activation, as well as reduces the density of dendritic spine in the hippocampus of APP/PS1 mice, while lentivirus-mediated inhibition of HDAC3 attenuates microglia activation and ameliorates cognition, as well as improves AD-related neuropathogenesis. These results indicate that inhibition of HDAC2 and HDAC3 can attenuate microglia activation and thus reverse the pathogenesis of AD. Correction of transcriptional dysregulation or inhibition of HDACs has been widely studied as a therapeutic strategy to reverse NDDs [129–132]. For instance, trichostatin A (TSA), one of the most recognized HDAC inhibitors, has been shown to exhibit effects of anti-inflammation and neuroprotection. Hsing et al. [129] have reported that TSA pretreatment attenuates microglia activation, reduces the production of inflammatory cytokines (e.g., TNF-*α*, MCP-1, and IL-1*β*), and improves cognitive function in mice and BV-2 cells. Meanwhile, TSA reduces A*β* plaques and oligomers by enhancing the phagocytosis of microglia and ameliorates cognitive function in APP/PS1 mice [131]. Comparably, suberoylanilide hydroxamic acid (SAHA), another pan-HDAC inhibitor, suppresses HDAC activity in microglia and reduces rotenone-induced inflammation and oxidative stress [133]. Additionally, valproic acid (VPA), a pan-HDAC inhibitor, is documented to exert neuroprotective actions in the rotenone rat model, paving the way for histone acetylation modifications of NDDs [134].

Simultaneously, other HDAC inhibitors have also exhibited therapeutic potential. Jiao et al. [135] have confirmed that CAY10683 (the HDAC2 inhibitor) also reduces levels of TNF-*α* and IL-1*β* in microglia via inhibiting the TLR4/NF-*κ*B signalling pathway. A recent study has demonstrated that WK2-16 (HDAC8 inhibitor) exerts neuroprotective effects by suppressing the expression of COX-2 and TNF-*α*, attenuating inflammatory response and microglia activation in vivo and in vitro [136]. In addition, it is demonstrated that MS-275 (HDAC inhibitor) ameliorates microglia activation and A*β* deposition in the APP/PS1 mice [137]. The sigma-1 receptor (Sig1R) plays an anti-inflammatory role in microglia, and its expression is reduced in the brains of NDD patients. A study by Iwamoto et al. [138] has shown that the HDAC6 inhibitor increases the expression Sig1R in primary microglia, implying that upregulation of Sig1R in microglia may be a strategy for the treatment of NDDs. Moreover, a subsequent study has shown that AGK2 (SIRT2 inhibitor) inhibits the expression of SIRT2, reduces microglia activation, corrects the imbalance of HATs/HDACs, and maintains DA neurons, playing a neuroprotective role in the prevention and treatment of PD [139]. A ChIP assay has shown that NaBu (HDAC inhibitor) enhances the expression of H3K9ac and promotes the transcription of PI3K, as well as facilitates AKT and CREB phosphorylation, which in turn upregulates the expression of BDNF and contributes to synaptic plasticity in BV-2 microglia [140].

Altogether, dysregulation of histone acetylations may be involved in the microglia-mediated neuroinflammation in NDDs, and thus, modulating the homeostasis of histone acetylation may be a novel promising approach for the treatment of NDDs. Most current studies have focused on the development of pan-HDAC inhibitors, while targeting specific HDAC isoforms or specific TFs correlated with NDDs is poorly understood. Additional studies are needed to identify the targeting drugs for histone acetylation.

#### 5.2.2. Histone Methylation

Methylation of histones is a covalent modification that occurs on arginine and lysine, resulting in the activation or repression of the gene expression, mainly by histone methyltransferases (HMTs). Generally, H3K4 trimethylation (H3K4me3) is implicated in the facilitation of transcription, whereas H3K9me3 leads to transcriptional repression. For instance, Ezh2 is an enzyme involved in the trimethylation of histone 3 lysine 27 (H3K27me3). Ezh2-mediated targeting of H3K27me3 inhibited the expression of Socs3, while Ezh2 deletion induced the upregulation of Socs3 and mediated ubiquitination of TRAF6, inhibiting the TLR/NF-*κ*B signalling pathway, which in turn attenuated microglia activation and inflammatory response [141]. In addition, a study by Matsuda et al. has revealed that [142] NeuroD1 (ND1) increases H3K27me3 levels and decreases H3K4me3 levels in specific promoter regions, as well as increases DNA methylation in microglia enhancer regions, suggesting that ND1 may attenuate microglia activation; the exact mechanism remains to be validated. Furthermore, Yang et al. [143] have confirmed that dextromethorphan (DM) treatment attenuates H3K4me3 modification of the TNF-*α* promoter gene locus in microglia and exerts anti-inflammatory effects, whereas the specific pathways or TFs involved remains to be verified. Jumonji domain containing 3 (Jmjd3), a histone H3K27me3 demethylase, and its suppression leads to aberrant activation of microglia and amplification of M1 phenotype, as well as exacerbating DA neuron loss in a mouse model of PD. It is possible that imbalance of M1/M2 microglia is responsible for accelerated neuronal death. Tang et al. [144] have disclosed reduced levels of Jmjd3, along with the elevated expression of H3K27me3 and higher ratios of microglia M1/M2 in aged mice, suggesting that upregulation of the Jmjd3 level may be able to facilitate the polarization of M2 phenotype by modifying H3K27me3, which may be an effective therapeutic method for the treatment of PD. Overall, histone methylation is implicated in the regulation of microglia and may involve multiple molecular mechanisms, with additional pathways or molecular mechanisms remaining to be clarified.

#### 5.2.3. Histone Phosphorylation and Sumoylation

There are fewer investigations into targeting histone sumoylation or phosphorylation in microglia, but the available evidence suggests that histone phosphorylation and sumoylation may be important factors in the progression of NDDs. Elevated histone H3 phospho(Ser10)-acetylation(Lys14) (H3S10phK14ac) may trigger the transcription of some inflammatory genes such as c-Fos, IL-6, and iNOS [145]. Sumoylation is a common protein translational modification that plays a role in mediating inflammatory response [146]. Phosphatidylinositol 3-kinase (PI3K) exerts an essential role in neuronal synaptic plasticity and inflammatory response via microglia and has been demonstrated to be phosphorylated [147]. Saw et al. [140] have revealed that expression of BDNF in microglia is subject to the sumoylation of PI3K as well as the PI3K/AKT pathway, highlighting that PI3K is an important upstream target involved in the epigenetic regulation of microglia and the enhancement of synaptic plasticity.

Taken together, aberrant activation of microglia induced by imbalance in histone modification homeostasis may contribute to the onset and progression of NDDs. Of these, studies of histone acetylation in NDDs have been widely reported. Regulation of histone acetylation homeostasis may facilitate physiological function of microglia and attenuate their aberrant activation or even reverse the pathological process of NDDs. HDACs play pivotal roles in the regulation of microglia function and dynamic homeostasis, and drug development for HDACs has achieved progress in NDDs [126]. Drug development targeting specific isoforms of HDACs may emerge as a therapeutic strategy to reverse NDDs.

### 5.3. Noncoding RNAs

Noncoding RNAs (ncRNAs) are types of a nonprotein-coding transcription factor that modulates cellular function with the aid of regulating gene expression, including microRNAs, circulating RNAs, and long ncRNAs [148]. MicroRNAs primarily drive the degradation of target mRNA, resulting in the transcriptional repression of genes [149]. Currently, dysregulation of microRNAs are extensively investigated in NDDs [150]. Cumulative evidence suggests that dysregulation of certain microRNAs contributes to excessive activation of microglia and chronic neuroinflammation [151]. As such, identification and modulation of specific microRNAs in NDDs may provide new perspectives for the treatment of NDDs.

Periyasamy et al. [152] have reported that the mechanism of HIV-1 Tat-mediated microglia activation is probably via downregulation of miR-124, and thus regulating the MECP2-STAT3 signaling axis, which may also be associated with DNA methylation of the miR-124 promoter. And promoting the expression miR-124 may be a way to suppress microglia hyperactivation. Consistent with this, studies by Yao et al. [153, 154] have revealed that exogenous delivery of miR-124 inhibits the expression of proinflammatory cytokines and attenuates microglia activation via the MEKK3/NF-*κ*B signalling pathway or suppresses the expression of p62/p38 in MPTP-induced PD mice. In addition, research on miR-124 has also been conducted in the N9 microglia cell line in vitro. The murine N9 microglia cell line, an immortalised cell line, is generated from microglia via retroviral-mediated transfection, which exhibits similar features to the primary cultured microglia, including phagocytosis and inflammation [155]. A study showing that translocation of miR-124 from mSOD1 MNs to exosomes may alter phenotypes of N9 microglia [156]. These results support that upregulation of miR-124 may attenuate microglia activation, ameliorate inflammation, and ultimately reverse the pathogenesis of NDDs. Meanwhile, miR-34a may damage phagocytosis by mediating the decreased expression of TREM2, leading to neuroinflammation and A*β* deposition [157]. Fenn et al. [158] have disclosed that elevated expression of age-related miR-29b is inversely correlated with the microglia regulators such as IGF-1 and CX3CL1. Notably, miR146 plays a negative regulatory role in innate immunity, while miR155 has the opposite role [100, 159]. Gupta et al. [160] have shown that miR-142-3p in microglia plays a key role in synaptic plasticity which may involve the CAMK2A-CREB-BDNF pathway. Furthermore, a study by Zhang et al. [161] has demonstrated that miR-711 inhibits the expression of Itpkb and tau phosphorylation, as well as increases the M2/M1 ratio, and ultimately improves cognitive function, suggesting that miR-711 may be an effective approach to alleviate NDDs.

Additionally, long ncRNAs (lncRNAs) have major roles in NDDs. Cai et al. [162] have demonstrated that long noncoding RNA metastasis-associated lung adenocarcinoma transcript 1(lncRNA MALAT1) epigenetically suppresses the expression of Nrf2 and triggers inflammatory response in PD mice and BV-2 cells. A novel lncRNA termed Nostrill is highly expressed in LPS-stimulated microglia cells with concomitant increased expression of iNOS. Silencing of Nostrill markedly decreased the expression of NF-*κ*B p65 and reduced activation of H3K4me3 at the iNOS site, as well as decreased neurotoxicity in mice and BV-2 cells [163]. LncRNA GAS5 acts as a regulator of microglia polarization, possibly by recruiting polycomb repressive complex 2 (PRC2), which represses transcription of the key factor TRF4 and thus promotes the conversion of microglia to M2 phenotype [164].

Taken together, accumulating evidence suggests that ncRNAs are closely linked to microglia and play an important role in the pathological process of NDDs. ncRNAs modulate a wide range of gene networks and are implicated in complex molecular mechanisms. Further studies on specific targets or molecular mechanisms of microRNAs are warranted in order to seek a potential strategy for the treatment of NDDs.

## 6. Conclusions

The pathogenesis of NDDs is complicated, with multiple pathological factors interspersed, leading to a vicious circle of pathological processes. Increasing evidence suggests that microglia have both favorable and detrimental roles in the occurrence and progression of NDDs [165]. With a better understanding of microglia, their involvement in NDDs has become a hot topic in this field. Neuroinflammation triggered by the abnormal activation of microglia is a distinctive hallmark of NDDs, and its potential mechanisms in these diseases remain to be elucidated. Hence, modulating function and phenotypes of microglia so as to reduce the production of pathogenic features may be a potential strategy to reverse NDDs.

**Figure 1:**
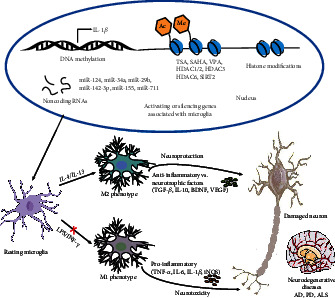
Possible mechanisms underlying epigenetic modulation of microglia in neurodegenerative diseases. Microglia-mediated neuroinflammation is a prominent feature of neurodegenerative diseases. Microglia exert neuroprotective and deleterious effects in neurodegenerative diseases that may be affected by their distinct phenotypes. Epigenetic mechanisms including DNA methylation, histone modifications, and noncoding RNAs are closely associated with neurodegenerative diseases. Dysregulated epigenetics on microglia-related genes may affect the function of microglia, resulting in neuroinflammation and neuronal damage, and exacerbating the progression of neurodegenerative diseases. Epigenetic modulation of microglia hyperactivation or their function and phenotypes, and consequently attenuating inflammatory responses and neuronal damage, may be a potential strategy to reverse neurodegenerative diseases.

With the rapid advances in modern technology, there has increasing evidence that microglia are epigenetically regulated, yet little is known about how epigenetic modulates microglia in NDDs. This updated review sheds light on different epigenetic mechanisms underlying the modulation of microglia in NDDs (Figure 1). It is clear that multiple epigenetic modifications are involved in the regulation of microglia function and phenotypes, yet several issues remain. Many of these epigenetic modifications are not directly linked to dynamic changes in microglia or phenotypes but rather focus more on the modulation of microglia function in NDDs. Additional studies on the epigenetic regulation of microglia phenotypes and function remain to be verified. In addition, the molecular mechanisms of epigenetic are complex and may involve integration between multiple epigenetic modifications. It is therefore particularly important to develop precisely targeted drugs for a specific locus. Last but not least, current studies are mostly focused on animal models, and extending research from animal models to humans remains a challenge, which requires high costs and advanced analytical techniques.

## References

[1] Adaikkan C., Middleton S. J., Marco A. (2019). Gamma entrainment binds higher-order brain regions and offers neuroprotection. *Neuron*.

[2] Cariccio V. L., Samà A., Bramanti P., Mazzon E. (2019). Mercury involvement in neuronal damage and in neurodegenerative diseases. *Biological trace element research*.

[3] Muddapu V. R., Dharshini S. A. P., Chakravarthy V. S., Gromiha M. M. (2020). Neurodegenerative diseases - is metabolic deficiency the root cause?. *Frontiers in neuroscience*.

[4] Tadokoro K., Ohta Y., Inufusa H., Loon A. F. N., Abe K. (2020). Prevention of cognitive decline in Alzheimer's disease by novel antioxidative supplements. *International journal of molecular sciences*.

[5] Hijaz B. A., Volpicelli-Daley L. A. (2020). Initiation and propagation of *α*-synuclein aggregation in the nervous system. *Molecular neurodegeneration*.

[6] Osaki T., Uzel S. G. M., Kamm R. D. (2018). Microphysiological 3D model of amyotrophic lateral sclerosis (ALS) from human iPS-derived muscle cells and optogenetic motor neurons. *Science advances*.

[7] Peng C., Trojanowski J. Q., Lee V. M.-Y. (2020). Protein transmission in neurodegenerative disease. *Nature reviews Neurology*.

[8] Niu X., Chen J., Gao J. (2019). Nanocarriers as a powerful vehicle to overcome blood-brain barrier in treating neurodegenerative diseases: focus on recent advances. *Asian journal of pharmaceutical sciences*.

[9] Chaves da Silva P. G., Hsu K., Benton J. L., Beltz B. S., Allodi S. (2020). A balancing act: the immune system supports neurodegeneration and neurogenesis. *Cellular and molecular neurobiology*.

[10] Fakhoury M. (2016). Immune-mediated processes in neurodegeneration: where do we stand?. *Journal of neurology*.

[11] Provenzano F., Pérez M. J., Deleidi M. (2021). Redefining microglial identity in health and disease at single-cell resolution. *Trends in molecular medicine*.

[12] Smith K. L., Kassem M. S., Clarke D. J. (2019). Microglial cell hyper-ramification and neuronal dendritic spine loss in the hippocampus and medial prefrontal cortex in a mouse model of PTSD. *Brain, behavior, and immunity*.

[13] Bernaus A., Blanco S., Sevilla A. (2020). Glia crosstalk in neuroinflammatory diseases. *Frontiers in cellular neuroscience*.

[14] Aldana B. I. (2019). Microglia-specific metabolic changes in neurodegeneration. *Journal of molecular biology*.

[15] Hickman S., Izzy S., Sen P., Morsett L., El Khoury J. (2018). Microglia in neurodegeneration. *Nature neuroscience*.

[16] Song W. M., Colonna M. (2018). The microglial response to neurodegenerative disease. *Advances in immunology*.

[17] Trotta T., Panaro M. A., Cianciulli A., Mori G., Di Benedetto A., Porro C. (2018). Microglia-derived extracellular vesicles in Alzheimer's Disease: A double- edged sword. *Biochemical pharmacology*.

[18] Sarlus H., Heneka M. T. (2017). Microglia in Alzheimer's disease. *The Journal of clinical investigation*.

[19] Neal M. L., Fleming S. M., Budge K. M. (2020). Pharmacological inhibition of CSF1R by GW2580 reduces microglial proliferation and is protective against neuroinflammation and dopaminergic neurodegeneration. *FASEB journal*.

[20] Subhramanyam C. S., Wang C., Hu Q., Dheen S. T. (2019). Microglia-mediated neuroinflammation in neurodegenerative diseases. *Seminars in cell & developmental biology*.

[21] Wu H., Chen Y., Zhu H., Zhao M., Lu Q. (2019). The pathogenic role of dysregulated epigenetic modifications in autoimmune diseases. *Frontiers in immunology*.

[22] Topper M. J., Vaz M., Marrone K. A., Brahmer J. R., Baylin S. B. (2020). The emerging role of epigenetic therapeutics in immuno-oncology. Nature reviews. *Clinical oncology*.

[23] Berson A., Nativio R., Berger S. L., Bonini N. M. (2018). Epigenetic regulation in neurodegenerative diseases. *Trends in neurosciences*.

[24] Bertogliat M. J., Morris-Blanco K. C., Vemuganti R. (2020). Epigenetic mechanisms of neurodegenerative diseases and acute brain injury. *Neurochemistry international*.

[25] Cheray M., Joseph B. (2018). Epigenetics control microglia plasticity. *Frontiers in cellular neuroscience*.

[26] Yeh H., Ikezu T. (2019). Transcriptional and epigenetic regulation of microglia in health and disease. *Trends in molecular medicine*.

[27] Nimmerjahn A., Kirchhoff F., Helmchen F. (2005). Resting microglial cells are highly dynamic surveillants of brain parenchyma in vivo. *Science*.

[28] Wu Y., Dissing-Olesen L., MacVicar B. A., Stevens B. (2015). Microglia: dynamic mediators of synapse development and plasticity. *Trends in immunology*.

[29] Vilalta A., Brown G. C. (2018). Neurophagy, the phagocytosis of live neurons and synapses by glia, contributes to brain development and disease. *The FEBS journal*.

[30] Andoh M., Ikegaya Y., Koyama R. (2019). Synaptic pruning by microglia in epilepsy. *Journal of clinical medicine*.

[31] Salter M. W., Beggs S. (2014). Sublime microglia: expanding roles for the guardians of the CNS. *Cell*.

[32] Sominsky L., De Luca S., Spencer S. J. (2018). Microglia: key players in neurodevelopment and neuronal plasticity. *The international journal of biochemistry & cell biology*.

[33] Augusto-Oliveira M., Arrifano G. P., Lopes-Araújo A. (2019). What do microglia really do in healthy adult brain?. *Cells*.

[34] Illes P., Rubini P., Ulrich H., Zhao Y., Tang Y. (2020). Regulation of microglial functions by purinergic mechanisms in the healthy and diseased CNS. *Cells*.

[35] Tsukahara T., Haniu H., Uemura T., Matsuda Y. (2020). Therapeutic potential of porcine liver decomposition product: new insights and perspectives for microglia-mediated neuroinflammation in neurodegenerative diseases. *Biomedicines*.

[36] Orihuela R., McPherson C. A., Harry G. J. (2016). Microglial M1/M2 polarization and metabolic states. *British journal of pharmacology*.

[37] Barati S., Kashani I. R., Moradi F. (2019). Mesenchymal stem cell mediated effects on microglial phenotype in cuprizone-induced demyelination model. *Journal of cellular biochemistry*.

[38] Sun G., Yang S., Cai H. (2019). Molybdenum disulfide nanoflowers mediated anti-inflammation macrophage modulation for spinal cord injury treatment. *Journal of colloid and interface science*.

[39] Shapouri-Moghaddam A., Mohammadian S., Vazini H. (2018). Macrophage plasticity, polarization, and function in health and disease. *Journal of cellular physiology*.

[40] Yang H. M., Yang S., Huang S. S., Tang B. S., Guo J. F. (2017). Microglial activation in the pathogenesis of Huntington's disease. *Frontiers in aging neuroscience*.

[41] Loane D. J., Kumar A. (2016). Microglia in the TBI brain: the good, the bad, and the dysregulated. *Experimental neurology*.

[42] Ren C., Li D., Zhou Q., Hu X. (2020). Mitochondria-targeted TPP-MoS(2) with dual enzyme activity provides efficient neuroprotection through M1/M2 microglial polarization in an Alzheimer's disease model. *Biomaterials*.

[43] Tang Y., Le W. (2016). Differential roles of M1 and M2 microglia in neurodegenerative diseases. *Molecular neurobiology*.

[44] Guzman-Martinez L., Maccioni R. B., Andrade V., Navarrete L. P., Pastor M. G., Ramos-Escobar N. (2019). Neuroinflammation as a common feature of neurodegenerative disorders. *Frontiers in pharmacology*.

[45] Beers D. R., Appel S. H. (2019). Immune dysregulation in amyotrophic lateral sclerosis: mechanisms and emerging therapies. *The Lancet Neurology*.

[46] Rajendran L., Paolicelli R. C. (2018). Microglia-mediated synapse loss in Alzheimer's disease. *The Journal of neuroscience*.

[47] Colonna M., Butovsky O. (2017). Microglia function in the central nervous system during health and neurodegeneration. *Annual review of immunology*.

[48] Zhang J., Zheng Y., Luo Y., Du Y., Zhang X., Fu J. (2019). Curcumin inhibits LPS-induced neuroinflammation by promoting microglial M2 polarization via TREM2/TLR4/NF-*κ*B pathways in BV2 cells. *Molecular immunology*.

[49] Kwon H. S., Koh S. H. (2020). Neuroinflammation in neurodegenerative disorders: the roles of microglia and astrocytes. *Translational neurodegeneration*.

[50] Martínez-Cué C., Rueda N. (2020). Cellular senescence in neurodegenerative diseases. *Frontiers in cellular neuroscience*.

[51] Kaur D., Sharma V., Deshmukh R. (2019). Activation of microglia and astrocytes: a roadway to neuroinflammation and Alzheimer's disease. *Inflammopharmacology*.

[52] Richardson J. R., Hossain M. M. (2013). Microglial Ion Channels as Potential Targets for Neuroprotection in Parkinson’s Disease. *Neural plasticity*.

[53] Lee E., Hwang I., Park S. (2019). MPTP-driven NLRP3 inflammasome activation in microglia plays a central role in dopaminergic neurodegeneration. *Cell death and differentiation*.

[54] Yao S., Li L., Sun X. (2019). FTY720 inhibits MPP^+^-induced microglial activation by affecting NLRP3 inflammasome activation. *Journal of neuroimmune pharmacology*.

[55] Tejera D., Mercan D., Sanchez‐Caro J. M. (2019). Systemic inflammation impairs microglial A*β* clearance through NLRP3 inflammasome. *The EMBO journal*.

[56] Haukedal H., Freude K. (2019). Implications of microglia in amyotrophic lateral sclerosis and frontotemporal dementia. *Journal of molecular biology*.

[57] Deora V., Lee J. D., Albornoz E. A. (2020). The microglial NLRP3 inflammasome is activated by amyotrophic lateral sclerosis proteins. *Glia*.

[58] Ermolaeva M., Neri F., Ori A., Rudolph K. L. (2018). Cellular and epigenetic drivers of stem cell ageing. *Nature reviews Molecular cell biology*.

[59] Wang T., Morency D. T., Harris N., Davis G. W. (2020). Epigenetic signaling in glia controls presynaptic homeostatic plasticity. *Neuron*.

[60] Nestler E. J., Lüscher C. (2019). The molecular basis of drug addiction: linking epigenetic to synaptic and circuit mechanisms. *Neuron*.

[61] Barter J. D., Foster T. C. (2018). Aging in the brain: new roles of epigenetics in cognitive decline. *The Neuroscientist : a review journal bringing neurobiology, neurology and psychiatry*.

[62] Roussel M. F., Stripay J. L. (2018). Epigenetic drivers in pediatric medulloblastoma. *Cerebellum*.

[63] Nativio R., Donahue G., Berson A. (2018). Dysregulation of the epigenetic landscape of normal aging in Alzheimer's disease. *Nature neuroscience*.

[64] Labbé C., Lorenzo-Betancor O., Ross O. A. (2016). Epigenetic regulation in Parkinson's disease. *Acta neuropathologica*.

[65] Talens R. P., Christensen K., Putter H. (2012). Epigenetic variation during the adult lifespan: cross-sectional and longitudinal data on monozygotic twin pairs. *Aging cell*.

[66] Jones M. J., Goodman S. J., Kobor M. S. (2015). DNA methylation and healthy human aging. *Aging cell*.

[67] Mohd Murshid N., Aminullah Lubis F., Makpol S. (2020). Epigenetic changes and its intervention in age-related neurodegenerative diseases. *Cellular and molecular neurobiology*.

[68] Monti N., Cavallaro R. A., Stoccoro A. (2020). CpG and non-CpG Presenilin1 methylation pattern in course of neurodevelopment and neurodegeneration is associated with gene expression in human and murine brain. *Epigenetics*.

[69] Feng J., Zhou Y., Campbell S. L. (2010). Dnmt1 and Dnmt3a maintain DNA methylation and regulate synaptic function in adult forebrain neurons. *Nature neuroscience*.

[70] Tong Z., Han C., Qiang M. (2015). Age-related formaldehyde interferes with DNA methyltransferase function, causing memory loss in Alzheimer's disease. *Neurobiology of aging*.

[71] Mastroeni D., Grover A., Delvaux E., Whiteside C., Coleman P. D., Rogers J. (2010). Epigenetic changes in Alzheimer's disease: decrements in DNA methylation. *Neurobiology of aging*.

[72] Chouliaras L., Mastroeni D., Delvaux E. (2013). Consistent decrease in global DNA methylation and hydroxymethylation in the hippocampus of Alzheimer's disease patients. *Neurobiology of aging*.

[73] Rocha E. M., De Miranda B., Sanders L. H. (2018). Alpha-synuclein: pathology, mitochondrial dysfunction and neuroinflammation in Parkinson's disease. *Neurobiology of disease*.

[74] Pihlstrøm L., Berge V., Rengmark A., Toft M. (2015). Parkinson's disease correlates with promoter methylation in the *α*-synuclein gene. *Movement disorders*.

[75] Kantor B., Tagliafierro L., Gu J. (2018). Downregulation of _SNCA_ Expression by Targeted Editing of DNA Methylation: A Potential Strategy for Precision Therapy in PD. *Molecular therapy*.

[76] Desplats P., Spencer B., Coffee E. (2011). *α*-Synuclein Sequesters Dnmt1 from the Nucleus:. *The Journal of biological chemistry*.

[77] Henderson-Smith A., Fisch K. M., Hua J. (2019). DNA methylation changes associated with Parkinson's disease progression: outcomes from the first longitudinal genome-wide methylation analysis in blood. *Epigenetics*.

[78] Martin L. J., Wong M. (2013). Aberrant regulation of DNA methylation in amyotrophic lateral sclerosis: a new target of disease mechanisms. *Neurotherapeutics*.

[79] Appleby‐Mallinder C., Schaber E., Kirby J. (2021). TDP43 proteinopathy is associated with aberrant DNA methylation in human amyotrophic lateral sclerosis. *Neuropathology and applied neurobiology*.

[80] Cobos S. N., Bennett S. A., Torrente M. P. (2019). The impact of histone post-translational modifications in neurodegenerative diseases. *Biochimica et biophysica acta Molecular basis of disease*.

[81] Saha R. N., Pahan K. (2006). HATs and HDACs in neurodegeneration: a tale of disconcerted acetylation homeostasis. *Cell death and differentiation*.

[82] Daskalaki M. G., Tsatsanis C., Kampranis S. C. (2018). Histone methylation and acetylation in macrophages as a mechanism for regulation of inflammatory responses. *Journal of cellular physiology*.

[83] Ganai S. A., Ramadoss M., Mahadevan V. (2016). Histone deacetylase (HDAC) Inhibitors - emerging roles in neuronal memory, learning, synaptic plasticity and neural regeneration. *Current neuropharmacology*.

[84] Lopez-Atalaya J. P., Barco A. (2014). Can changes in histone acetylation contribute to memory formation?. *Trends in genetics*.

[85] Francis Y. I., Fa M., Ashraf H. (2009). Dysregulation of histone acetylation in the APP/PS1 mouse model of Alzheimer's disease. *Journal of Alzheimer's disease*.

[86] Schueller E., Paiva I., Blanc F. (2020). Dysregulation of histone acetylation pathways in hippocampus and frontal cortex of Alzheimer's disease patients. *European neuropsychopharmacology*.

[87] Peleg S., Sananbenesi F., Zovoilis A. (2010). Altered histone acetylation is associated with age-dependent memory impairment in mice. *Science*.

[88] Gräff J., Rei D., Guan J. S. (2012). An epigenetic blockade of cognitive functions in the neurodegenerating brain. *Nature*.

[89] Marzi S. J., Leung S. K., Ribarska T. (2018). A histone acetylome-wide association study of Alzheimer's disease identifies disease-associated H3K27ac differences in the entorhinal cortex. *Nature neuroscience*.

[90] Harrison I. F., Dexter D. T. (2013). Epigenetic targeting of histone deacetylase: therapeutic potential in Parkinson's disease?. *Pharmacology & therapeutics*.

[91] Park G., Tan J., Garcia G., Kang Y., Salvesen G., Zhang Z. (2016). Regulation of Histone Acetylation by Autophagy in Parkinson Disease. *The Journal of biological chemistry*.

[92] Kontopoulos E., Parvin J. D., Feany M. B. (2006). Alpha-synuclein acts in the nucleus to inhibit histone acetylation and promote neurotoxicity. *Human molecular genetics*.

[93] Shukla S., Tekwani B. L. (2020). Histone deacetylases Inhibitors in neurodegenerative diseases, neuroprotection and neuronal differentiation. *Frontiers in pharmacology*.

[94] Gupta R., Ambasta R. K., Kumar P. (2020). Pharmacological intervention of histone deacetylase enzymes in the neurodegenerative disorders. *Life sciences*.

[95] Hampel H., Vassar R., De Strooper B. (2021). The *β*-secretase BACE1 in Alzheimer's disease. *Biological psychiatry*.

[96] Hébert S. S., Horré K., Nicolaï L. (2008). Loss of microRNA cluster miR-29a/b-1 in sporadic Alzheimer's disease correlates with increased BACE1/beta-secretase expression. *Proceedings of the National Academy of Sciences of the United States of America*.

[97] Li P., Xu Y., Wang B., Huang J., Li Q. (2020). miR-34a-5p and miR-125b-5p attenuate A*β*-induced neurotoxicity through targeting BACE1. *Journal of the neurological sciences*.

[98] Li J., Chen W., Yi Y., Tong Q. (2019). miR-219-5p inhibits tau phosphorylation by targeting TTBK1 and GSK-3*β* in Alzheimer's disease. *Journal of cellular biochemistry*.

[99] Doxakis E. (2010). Post-transcriptional Regulation of *α*-Synuclein Expression by mir-7 and mir-153. *The Journal of biological chemistry*.

[100] Thome A. D., Harms A. S., Volpicelli-Daley L. A., Standaert D. G. (2016). microRNA-155 regulates alpha-synuclein-induced inflammatory responses in models of Parkinson disease. *The Journal of neuroscience*.

[101] Bennett S. A., Tanaz R., Cobos S. N., Torrente M. P. (2019). Epigenetics in amyotrophic lateral sclerosis: a role for histone post- translational modifications in neurodegenerative disease. *Translational research*.

[102] Lewcock J. W., Schlepckow K., Di Paolo G., Tahirovic S., Monroe K. M., Haass C. (2020). Emerging microglia biology defines novel therapeutic approaches for Alzheimer's disease. *Neuron*.

[103] Holtman I. R., Skola D., Glass C. K. (2017). Transcriptional control of microglia phenotypes in health and disease. *The Journal of clinical investigation*.

[104] Martins-Ferreira R., Leal B., Costa P. P., Ballestar E. (2021). Microglial innate memory and epigenetic reprogramming in neurological disorders. *Progress in neurobiology*.

[105] Das A., Chai J. C., Kim S. H. (2015). Dual RNA sequencing reveals the expression of unique transcriptomic signatures in lipopolysaccharide-induced BV-2 microglial cells. *PloS one*.

[106] Cao M., Cortes M., Moore C. S. (2015). Fetal microglial phenotype in vitro carries memory of prior in vivo exposure to inflammation. *Frontiers in cellular neuroscience*.

[107] Mota M., Porrini V., Parrella E. (2020). Neuroprotective epi-drugs quench the inflammatory response and microglial/macrophage activation in a mouse model of permanent brain ischemia. *Journal of neuroinflammation*.

[108] Razin A., Cedar H. (2010). DNA methylation and gene expression. *Microbiology and Molecular Biology Reviews*.

[109] Wüllner U., Kaut O., de Boni L., Piston D., Schmitt I. (2016). DNA methylation in Parkinson's disease. *Journal of neurochemistry*.

[110] Keleshian V. L., Modi H. R., Rapoport S. I., Rao J. S. (2013). Aging is associated with altered inflammatory, arachidonic acid cascade, and synaptic markers, influenced by epigenetic modifications, in the human frontal cortex. *Journal of neurochemistry*.

[111] Jay T. R., von Saucken V. E., Landreth G. E. (2017). TREM2 in neurodegenerative diseases. *Molecular neurodegeneration*.

[112] Celarain N., De Gordoa J. S., Zelaya M. V. (2016). TREM2 upregulation correlates with 5-hydroxymethycytosine enrichment in Alzheimer's disease hippocampus. *Clinical epigenetics*.

[113] Byun C. J., Seo J., Jo S. A. (2012). DNA methylation of the 5′-untranslated region at +298 and +351 represses BACE1 expression in mouse BV-2 microglial cells. *Biochemical and biophysical research communications*.

[114] Prati F., De Simone A., Armirotti A. (2015). 3,4-Dihydro-1,3,5-triazin-2(1H)-ones as the first dual BACE-1/GSK-3*β* fragment hits against Alzheimer's disease. *ACS chemical neuroscience*.

[115] Lin H. C., Hsieh H. M., Chen Y. H., Hu M. L. (2009). _S_ -Adenosylhomocysteine increases *β*-amyloid formation in BV-2 microglial cells by increased expressions of *β*-amyloid precursor protein and presenilin 1 and by hypomethylation of these gene promoters. *Neurotoxicology*.

[116] Zhang C. J., Jiang M., Zhou H. (2018). TLR-stimulated IRAKM activates caspase-8 inflammasome in microglia and promotes neuroinflammation. *The Journal of clinical investigation*.

[117] Libert S., Guarente L. (2013). Metabolic and neuropsychiatric effects of calorie restriction and sirtuins. *Annual review of physiology*.

[118] Zhang Z., Shen Q., Wu X., Zhang D., Xing D. (2020). Activation of PKA/SIRT1 signaling pathway by photobiomodulation therapy reduces A*β* levels in Alzheimer's disease models. *Aging cell*.

[119] Cho S. H., Chen J. A., Sayed F. (2015). SIRT1 deficiency in microglia contributes to cognitive decline in aging and neurodegeneration via epigenetic regulation of IL-1*β*. *The Journal of neuroscience*.

[120] Matt S. M., Lawson M. A., Johnson R. W. (2016). Aging and peripheral lipopolysaccharide can modulate epigenetic regulators and decrease IL-1*β* promoter DNA methylation in microglia. *Neurobiology of aging*.

[121] Sanchez-Mut J. V., Heyn H., Vidal E. (2016). Human DNA methylomes of neurodegenerative diseases show common epigenomic patterns. *Translational psychiatry*.

[122] Gijselinck I., Van Mossevelde S., van der Zee J. (2016). The _C9orf72_ repeat size correlates with onset age of disease, DNA methylation and transcriptional downregulation of the promoter. *Molecular psychiatry*.

[123] Venkatesh S., Workman J. L. (2015). Histone exchange, chromatin structure and the regulation of transcription. *Nature reviews Molecular cell biology*.

[124] Chen K., Bennett S. A., Rana N. (2018). Neurodegenerative disease proteinopathies are connected to distinct histone post-translational modification landscapes. *ACS chemical neuroscience*.

[125] Penney J., Tsai L. H. (2014). Histone deacetylases in memory and cognition. *Science signaling*.

[126] Datta M., Staszewski O., Raschi E. (2018). Histone deacetylases 1 and 2 regulate microglia function during development, homeostasis, and neurodegeneration in a context-dependent manner. *Immunity*.

[127] Sun X. Y., Zheng T., Yang X. (2019). HDAC2 hyperexpression alters hippocampal neuronal transcription and microglial activity in neuroinflammation-induced cognitive dysfunction. *Journal of neuroinflammation*.

[128] Zhu X., Wang S., Yu L. (2017). HDAC3 negatively regulates spatial memory in a mouse model of Alzheimer's disease. *Aging cell*.

[129] Hsing C. H., Hung S. K., Chen Y. C. (2015). Histone deacetylase inhibitor trichostatin A ameliorated endotoxin-induced neuroinflammation and cognitive dysfunction. *Mediators of inflammation*.

[130] Alqinyah M., Maganti N., Ali M. W. (2017). Regulator of G protein signaling 10 (Rgs10) expression is transcriptionally silenced in activated microglia by histone deacetylase activity. *Molecular pharmacology*.

[131] Su Q., Li T., He P. F. (2021). Trichostatin A ameliorates Alzheimer's disease-related pathology and cognitive deficits by increasing albumin expression and A*β* clearance in APP/PS1 mice. *Alzheimer's research & therapy*.

[132] Nakamura Y., Kimura S., Takada N. (2020). Stimulation of toll-like receptor 4 downregulates the expression of *α*7 nicotinic acetylcholine receptors via histone deacetylase in rodent microglia. *Neurochemistry international*.

[133] Günaydin C., Çelik Z. B., Bilge S. S., Avci B., Kara N. (2021). SAHA attenuates rotenone-induced toxicity in primary microglia and HT-22 cells. *Toxicology and industrial health*.

[134] Monti B., Gatta V., Piretti F., Raffaelli S. S., Virgili M., Contestabile A. (2010). Valproic acid is neuroprotective in the rotenone rat model of Parkinson's disease: involvement of alpha-synuclein. *Neurotoxicity research*.

[135] Jiao F. Z., Wang Y., Zhang H. Y., Zhang W. B., Wang L. W., Gong Z. J. (2018). Histone deacetylase 2 inhibitor CAY10683 alleviates lipopolysaccharide induced neuroinflammation through attenuating TLR4/NF-*κ*B signaling pathway. *Neurochemical research*.

[136] Lin F. L., Yen J. L., Kuo Y. C. (2019). HADC8 inhibitor WK2-16 therapeutically targets lipopolysaccharide-induced mouse model of neuroinflammation and microglial activation. *International journal of molecular sciences*.

[137] Zhang Z. Y., Schluesener H. J. (2013). Oral administration of histone deacetylase inhibitor MS-275 ameliorates neuroinflammation and cerebral amyloidosis and improves behavior in a mouse model. *Journal of neuropathology and experimental neurology*.

[138] Iwamoto M., Nakamura Y., Takemura M., Hisaoka-Nakashima K., Morioka N. (2020). TLR4-TAK1-p38 MAPK pathway and HDAC6 regulate the expression of sigma-1 receptors in rat primary cultured microglia. *Journal of pharmacological sciences*.

[139] Harrison I. F., Smith A. D., Dexter D. T. (2018). Pathological histone acetylation in Parkinson's disease: neuroprotection and inhibition of microglial activation through SIRT 2 inhibition. *Neuroscience letters*.

[140] Saw G., Krishna K., Gupta N. (2020). Epigenetic regulation of microglial phosphatidylinositol 3-kinase pathway involved in long-term potentiation and synaptic plasticity in rats. *Glia*.

[141] Zhang X., Wang Y., Yuan J. (2018). Macrophage/microglial Ezh2 facilitates autoimmune inflammation through inhibition of Socs3. *The Journal of experimental medicine*.

[142] Matsuda T., Irie T., Katsurabayashi S. (2019). Pioneer factor neuroD1 rearranges transcriptional and epigenetic profiles to execute microglia-neuron conversion. *Neuron*.

[143] Yang Y. N., Yang Y. S. H., Wu P. L., Yang C. H., Kuo K. C., Yang S. N. (2020). Dextromethorphan suppresses lipopolysaccharide-induced epigenetic histone regulation in the tumor necrosis factor-*α* expression in primary rat microglia. *Mediators of inflammation*.

[144] Tang Y., Li T., Li J. (2014). Jmjd3 is essential for the epigenetic modulation of microglia phenotypes in the immune pathogenesis of Parkinson's disease. *Cell death and differentiation*.

[145] Rigillo G., Vilella A., Benatti C. (2018). LPS-induced histone H3 phospho(Ser10)-acetylation(Lys14) regulates neuronal and microglial neuroinflammatory response. *Brain, behavior, and immunity*.

[146] Rigillo G., Vilella A., Benatti C. (2005). A SUMOylation-dependent pathway mediates transrepression of inflammatory response genes by PPAR-*γ*. *Nature*.

[147] De la Cruz-Herrera C. F., Baz-Martínez M., Lang V. (2016). Conjugation of SUMO to p85 leads to a novel mechanism of PI3K regulation. *Oncogene*.

[148] Morris K. V., Mattick J. S. (2014). The rise of regulatory RNA. *Nature reviews Genetics*.

[149] Jonas S., Izaurralde E. (2015). Towards a molecular understanding of microRNA-mediated gene silencing. *Nature reviews Genetics*.

[150] Juźwik C. A., Drake S. S., Zhang Y. (2019). MicroRNA dysregulation in neurodegenerative diseases: a systematic review. *Progress in neurobiology*.

[151] Guo Y., Hong W., Wang X. (2019). MicroRNAs in microglia: how do microRNAs affect activation, inflammation, polarization of microglia and mediate the interaction between microglia and glioma?. *Frontiers in molecular neuroscience*.

[152] Periyasamy P., Thangaraj A., Guo M. L., Hu G., Callen S., Buch S. (2018). Epigenetic Promoter DNA Methylation of miR-124 promotes HIV-1 Tat-mediated microglial activation via MECP2-STAT3 axis. *The Journal of neuroscience*.

[153] Yao L., Ye Y., Mao H. (2018). MicroRNA-124 regulates the expression of MEKK3 in the inflammatory pathogenesis of Parkinson's disease. *Journal of neuroinflammation*.

[154] Yao L., Zhu Z., Wu J. (2019). MicroRNA-124 regulates the expression of p62/p38 and promotes autophagy in the inflammatory pathogenesis of Parkinson's disease. *FASEB journal*.

[155] Fleisher-Berkovich S., Filipovich-Rimon T., Ben-Shmuel S., Hülsmann C., Kummer M. P., Heneka M. T. (2010). Distinct modulation of microglial amyloid beta phagocytosis and migration by neuropeptides. *Journal of neuroinflammation*.

[156] Pinto S., Cunha C., Barbosa M., Vaz A. R., Brites D. (2017). Exosomes from NSC-34 Cells transfected with hSOD1-G93A are enriched in miR-124 and drive alterations in microglia phenotype. *Frontiers in neuroscience*.

[157] Alexandrov P. N., Zhao Y., Jones B. M., Bhattacharjee S., Lukiw W. J. (2013). Expression of the phagocytosis-essential protein TREM2 is down-regulated by an aluminum-induced miRNA-34a in a murine microglial cell line. *Journal of inorganic biochemistry*.

[158] Fenn A. M., Smith K. M., Lovett-Racke A. E., Guerau-de-Arellano M., Whitacre C. C., Godbout J. P. (2013). Increased micro-RNA 29b in the aged brain correlates with the reduction of insulin-like growth factor-1 and fractalkine ligand. *Neurobiology of aging*.

[159] Jayadev S., Case A., Alajajian B., Eastman A. J., Möller T., Garden G. A. (2013). Presenilin 2 influences miR146 level and activity in microglia. *Journal of neurochemistry*.

[160] Gupta N., Jadhav S., Tan K. L., Saw G., Mallilankaraman K. B., Dheen S. T. (2020). miR-142-3p regulates BDNF expression in activated rodent microglia through its target CAMK2A. *Frontiers in cellular neuroscience*.

[161] Zhang Y., Xu C., Nan Y., Nan S. (2020). Microglia-derived extracellular vesicles carrying miR-711 alleviate neurodegeneration in a murine Alzheimer's disease model by binding to Itpkb. *Frontiers in cell and developmental biology*.

[162] Cai L. J., Tu L., Huang X. M. (2020). lncRNA MALAT1 facilitates inflammasome activation via epigenetic suppression of Nrf2 in Parkinson's disease. *Molecular brain*.

[163] Mathy N. W., Burleigh O., Kochvar A. (2021). A novel long intergenic non-coding RNA, Nostrill, regulates iNOS gene transcription and neurotoxicity in microglia. *Journal of neuroinflammation*.

[164] Sun D., Yu Z., Fang X. (2017). lncRNA GAS5 inhibits microglial M2 polarization and exacerbates demyelination. *EMBO reports*.

[165] Anwar S., Rivest S. (2020). Alzheimer's disease: microglia targets and their modulation to promote amyloid phagocytosis and mitigate neuroinflammation. *Expert opinion on therapeutic targets*.

